# Reproducibility of a peripheral quantitative computed tomography scan protocol to measure the material properties of the second metatarsal

**DOI:** 10.1186/1471-2474-15-242

**Published:** 2014-07-19

**Authors:** Elodie Chaplais, David Greene, Anita Hood, Scott Telfer, Verona du Toit, Davinder Singh-Grewal, Joshua Burns, Keith Rome, Daniel J Schiferl, Gordon J Hendry

**Affiliations:** 1 School of Exercise Science, Australian Catholic University, Strathfield, NSW, Australia; 2 Clermont Université, Laboratoire des Adaptations Métaboliques à l'Exercice en Conditions Physiologiques et Pathologiques, Clermont-Ferrand, France; 3 Podiatry Department, Concord Hospital, South Western Sydney Local Health District, Sydney, NSW, Australia; 4 Institute for Applied Health Research, Glasgow Caledonian University, Glasgow G4 0BA, UK; 5 School of Medicine, University of Western Sydney, Penrith, NSW, Australia; 6 The University of Sydney Discipline of Paediatrics and Child Health, Sydney, NSW, Australia; 7 The University of New South Wales School of Women and Children’s Health, Sydney, NSW, Australia; 8 The Sydney Children’s Hospitals Network Randwick and Westmead Department of Rheumatology, Sydney, NSW, Australia; 9 The University of Sydney and Sydney Children’s Hospitals Network (Randwick and Westmead), Sydney, NSW, Australia; 10 Department of Podiatry, Health and Rehabilitation Research Institute, School of Rehabilitation & Occupational Studies, AUT, Auckland, New Zealand; 11 Bone Diagnostic Inc, Fort Atkinson, WI, USA; 12 School of Science & Health, University of Western Sydney, Penrith, NSW, Australia

**Keywords:** pQCT, Fracture risk, 2^nd^ metatarsal, Foot, Osteoporosis, Insufficiency fracture

## Abstract

**Background:**

Peripheral quantitative computed tomography (pQCT) is an established technology that allows for the measurement of the material properties of bone. Alterations to bone architecture are associated with an increased risk of fracture. Further pQCT research is necessary to identify regions of interest that are prone to fracture risk in people with chronic diseases. The second metatarsal is a common site for the development of insufficiency fractures, and as such the aim of this study was to assess the reproducibility of a novel scanning protocol of the second metatarsal using pQCT.

**Methods:**

Eleven embalmed cadaveric leg specimens were scanned six times; three times with and without repositioning. Each foot was positioned on a custom-designed acrylic foot plate to permit unimpeded scans of the region of interest. Sixty-six scans were obtained at 15% (distal) and 50% (mid shaft) of the second metatarsal. Voxel size and scan speed were reduced to 0.40 mm and 25 mm.sec^-1^. The reference line was positioned at the most distal portion of the 2^nd^ metatarsal. Repeated measurements of six key variables related to bone properties were subject to reproducibility testing. Data were log transformed and reproducibility of scans were assessed using intraclass correlation coefficients (ICC) and coefficients of variation (CV%).

**Results:**

Reproducibility of the measurements without repositioning were estimated as: trabecular area (ICC 0.95; CV% 2.4), trabecular density (ICC 0.98; CV% 3.0), Strength Strain Index (SSI) - distal (ICC 0.99; CV% 5.6), cortical area (ICC 1.0; CV% 1.5), cortical density (ICC 0.99; CV% 0.1), SSI – mid shaft (ICC 1.0; CV% 2.4). Reproducibility of the measurements after repositioning were estimated as: trabecular area (ICC 0.96; CV% 2.4), trabecular density (ICC 0.98; CV% 2.8), SSI - distal (ICC 1.0; CV% 3.5), cortical area (ICC 0.99; CV%2.4), cortical density (ICC 0.98; CV% 0.8), SSI – mid shaft (ICC 0.99; CV% 3.2).

**Conclusions:**

The scanning protocol generated excellent reproducibility for key bone properties measured at the distal and mid-shaft regions of the 2^nd^ metatarsal. This protocol extends the capabilities of pQCT to evaluate bone quality in people who may be at an increased risk of metatarsal insufficiency fractures.

## Background

Insufficiency stress fractures are mal-adaptations of poor quality bone in the form of deficient elastic resistance in response to normal muscle strain, and these can result in significant levels of morbidity [1,2]. The 2nd metatarsal is one of the most common sites of insufficiency fracture in people with poor bone strength, whereas fatigue-type stress fractures in athletes and military recruits which are characterized by abnormal stresses being applied to bone which has normal elastic resistance due to muscle fatigue [2-6]. The reasons for the predilection of fractures at the 2^nd^ metatarsal remain unclear; however the research evidence suggests that the 2^nd^ metatarsal may be prone to greater levels of bone strain relative to the lesser metatarsals [3,7]. Indeed, the maximum tensile stress experienced in the 2^nd^ metatarsal is at the mid shaft, typically the narrowest portion of the bone and the portion most prone to fracture [3,8]. Bone strength of the 2^nd^ metatarsal appears to be closely related to cortical bone mineral density [8]. As such, the structural integrity of the 2nd metatarsal may be diminished as a result of exposure to disease-related risk factors that affect bone strength. However it is acknowledged that the complex relationship between bone mineral density and fracture risk remains unclear and requires further investigation [9].

Bone strength can be estimated from measurements of bone mineral density (BMD) [8]. Peripheral quantitative computed tomography (pQCT) is a three-dimensional imaging technology that assesses trabecular and cortical bone characteristics, including volumetric BMD (vBMD), at peripheral sites such as the tibia and radius [10]. Peripheral quantitative computed tomography (pQCT) has emerged as an accurate method for measuring BMD and is advantageous as it is less susceptible to confounding by skeletal size compared to dual x-ray absorptiometry (DXA) [11]. Furthermore, pQCT scans are relatively safe as the level of radiation exposure is low [10,12].

The International Society of Clinical Densitometry has highlighted the need for further pQCT research to determine other regions of interest (ROI) that are predictive of fracture risk in people with chronic diseases [12]. Currently there is no gold standard method to measure bone quality in the foot. Standard protocols for pQCT measurements of the peripheral appendicular skeleton are largely limited to the wrist and tibia [10]. However bone density measurements in the foot may correlate only slightly with more proximal sites [13]. As such, there is a need for a reproducible protocol for pQCT BMD measurements of the 2nd metatarsal. Good reproducibility and precision of high resolution pQCT measurements of the anthropometrically similar metacarpals has been demonstrated recently in people with and without rheumatoid arthritis [14,15]. If a standard, reproducible protocol for measuring 2nd metatarsal bone health using pQCT can be developed, it may be possible to identify people who are at risk of developing insufficiency fractures at this ROI. Accordingly, our aim was to assess the reproducibility of a novel scanning protocol to measure the material properties of the 2^nd^ metatarsal using pQCT.

## Methods

### Subjects

Prior to receipt of donated bodies for teaching and/or scientific purposes at the University of Western Sydney Anatomy Department of the School of Science & Health, consent is established using a Body Consent Form. Eleven male adult embalmed cadaver legs (4 right and 7 left legs), disarticulated at the hip, were made available by the University of Western Sydney Anatomy Department of the School of Science & Health, and scanned with soft tissue intact. Specimens had been embalmed using a chemical mixture of 70% ethanol, glycerol, 40% formaldehyde and water. Each specimen was refrigerated for a minimum of 12 months prior to disarticulation. Age at death ranged from 59 to 93 years with a mean age at death of 75 years. Scans were performed at the Anatomy Department at the School of Science & Health, University of Western Sydney, Campbelltown campus. Ethical approval was granted by the University of Western Sydney Research Ethics Committee in July, 2012. This research was conducted in accordance with the Declaration of Helsinki.

### Experimental scanning protocol

An experienced pQCT operator (DG) performed all pQCT scans using a Stratec XCT 2000 device (Stratec Medizintechnik GmbH, Pforzeim, Germany, software version 5.50d). A cone phantom was scanned at the commencement of data collection to confirm machine calibration. Briefly, the cone phantom used to calibrate the pQCT scanner was the Quality Assurance in Radiology and Medicine European Forearm Phantom (QRM-EFP), which consists of water- and bone-equivalent solid materials and is a standardized device that is used to test peripheral bone densitometry systems [16]. The pQCT scanner was calibrated on a daily basis. All scans were acquired over a two day period, and the pQCT scanner was calibrated prior to the commencement of scans each day. The effective dose of radiation for this study was calculated by an independent radiation safety officer as 0.0035 millisieverts (mSv), which is within the dose constraints for children and adults and represents a negligible risk [17].

**Figure 1:**
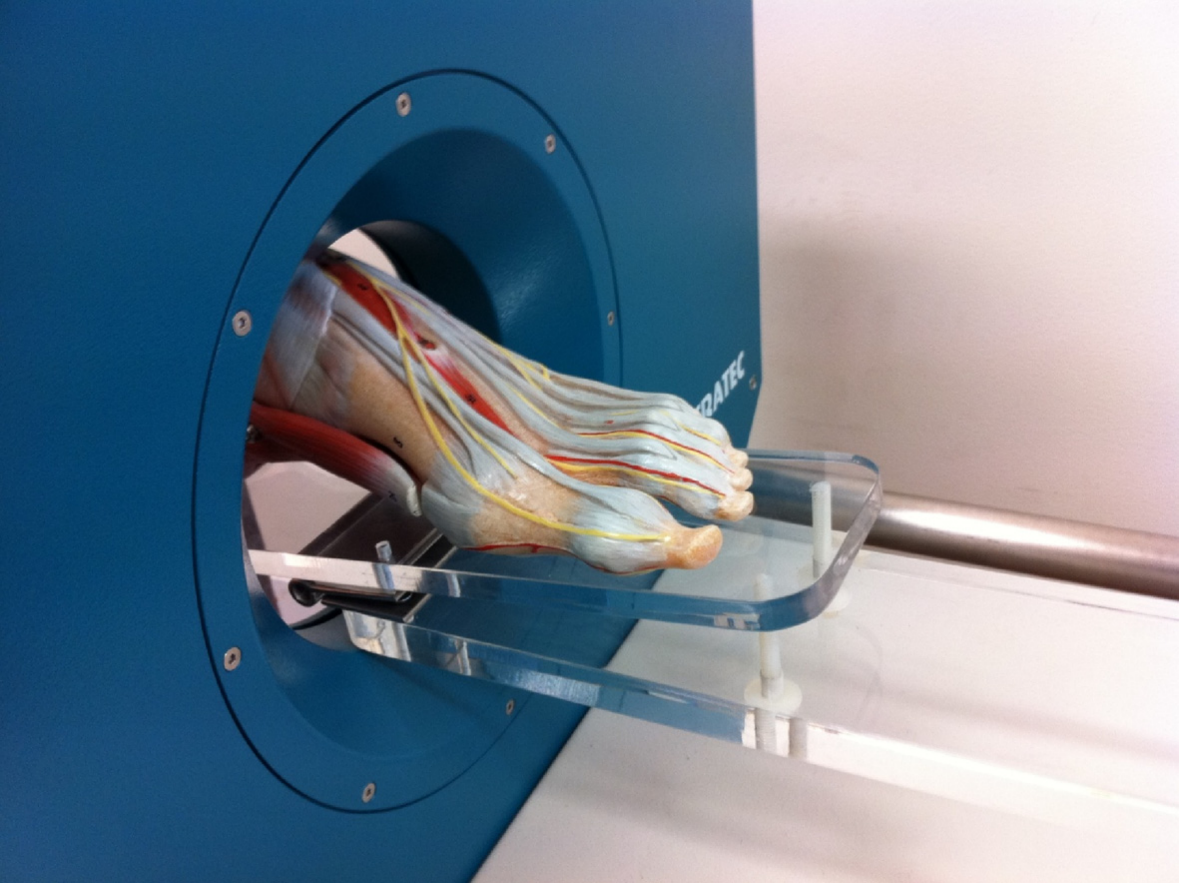
Custom designed acrylic foot plate and foot model illustrating the experimental pQCT protocol.

**Figure 2:**
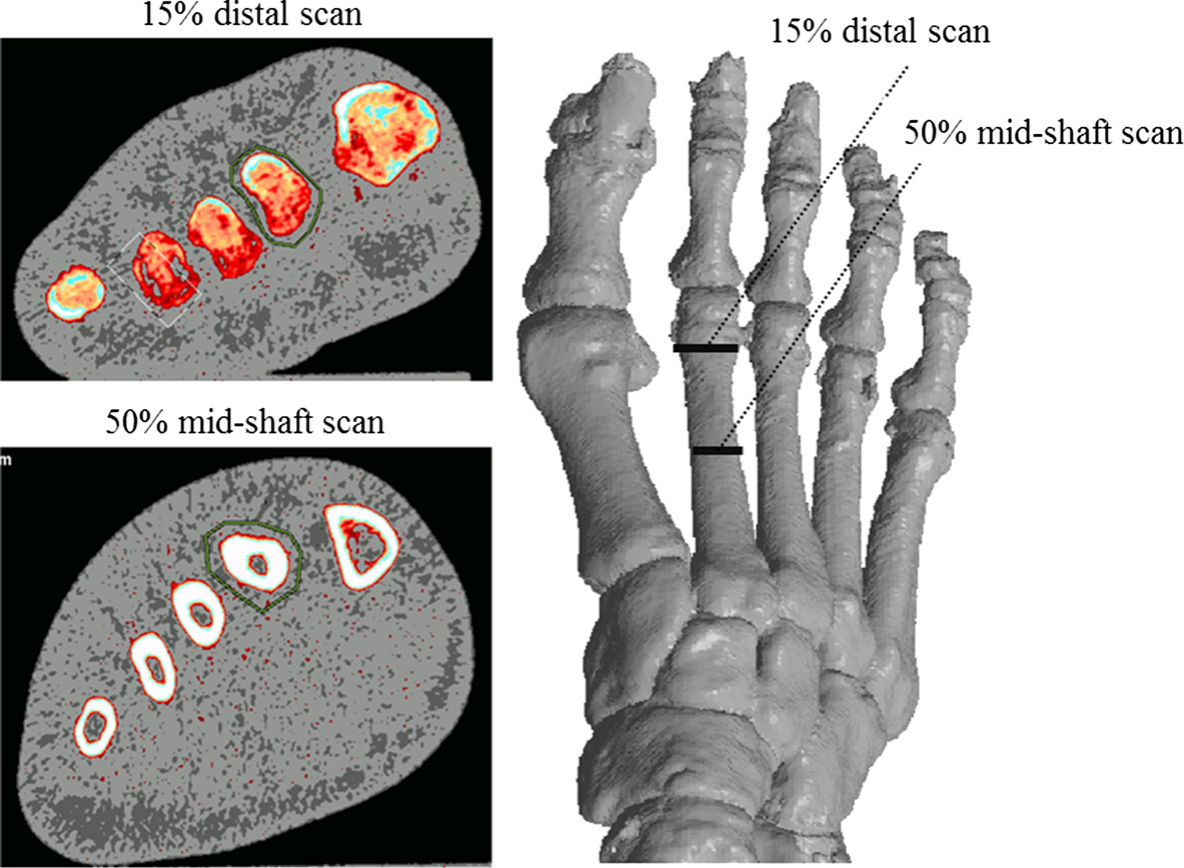
**Illustration depicting the scout images and regions of interest of the 2**^**nd **^**metatarsal bone.**

Each foot was positioned on a custom-designed foot plate comprised of acrylic to allow the pQCT gantry to pass over the foot scan-region unimpeded (Figure 1). The foot was strapped to the foot plate at the ankle and toes. A scout scan of the entire 2^nd^ metatarsal was performed to assess metatarsal length and to provide an anatomic reference line. The reference line was positioned at the most distal portion of the 2^nd^ metatarsal. Sixty-six scans (n = 66) were obtained at 15% (distal end) and 50% (mid shaft) of the 2^nd^ metatarsal (Figure 2). As single scans were acquired at these sites, no averaging of multiple slices was required. Each slice obtained was 2.2 mm wide.

Each foot was scanned six times; three times with, and three times without repositioning over a period of two days. Voxel size and scan speed were set to 0.40 mm and 25 mm.sec^-1^, respectively. Because the 15% scan site has very little cortical shell, we suspected that a standard contour search would not be reliable due to the cortical shell being thinner than the 0.4 mm voxel size. Normal interactive contour search modes 2 and 3 would therefore fail to detect bone. Contour mode 1 is a threshold based algorithm used at a density of 0 mg/cm^3^ so all voxels could be included within the ROI to describe total bone. Therefore the ROI was drawn to define the periosteal edge, allowing contour mode 1 and a threshold level of zero to detect total bone. Peel mode 4 with a threshold of 650 and 1% peel back was used to define the trabecular area. Peel mode 4 is a combination of the threshold based peel mode 2 with an additional peel back based on a percentage of total bone. The method was adopted to purposely use a high threshold of 650 mg/cm^3^ so all possible trabecular bone was detected. This analysis mode then ‘peels back’ from the threshold found endosteum so that any cortical bone detections are removed. At 50% mid shaft, a 600 mg.cm^3^ threshold and separation mode 2 were employed. The pQCT variables selected for repeat measurement included strength strain index (SSI, mm^3^), cortical area (mm^2^), cortical density (g.cm^2^), trabecular area (mm^2^), and trabecular density (g.cm^2^). A single rater (DG) analysed all scans in one sitting.

### Statistical analysis

Statistical analyses were performed using SPSS version 20.0 (SPSS, Chicago, IL, USA). Intra-class correlation (ICC) was calculated using a two-tailed mixed consistency model [18]. Data were then natural log transformed to meet the assumptions of inferential statistical analyses and coefficient of variations (CV%s) calculated using mean square error (MSE) and expressed as a percentage (CV%). An ICC of 1.0 indicates perfect agreement and an ICC of >0.75 represents “excellent reliability” [19]. CV values of 10% or below are generally considered to be an indication of “good” reliability [20]. Means and standard deviations (SD) are presented for unadjusted values.

## Results and discussion

Table 1 shows unadjusted means and SDs, and log transformed ICC and CV% values. ICC analysis revealed excellent reproducibility for scans with, and without, repositioning. Without repositioning, ICC ranged from 0.95 to 1.0. After repositioning, ICC ranged from 0.96 to 1.0. When all scans were analysed, ICC ranged from 0.95 to 1.0. CV% results also indicate adequate reproducibility ranging from a minimum value of 0.1 to 5.6 for scans without repositioning and 0.8 to 3.5 after repositioning. When all scans were analysed, CV% ranged from 0.5 to 8.9.

**Table 1:** Mean & SD (unadjusted vales), ICC and CV% (log transformed) for scans with and without repositioning and all scans combined

		**Without repositioning (n = 33)**				**With repositioning (n = 33)**					**All scans (n = 66)**			
**Site**	**Variable**	**Mean ± SD**	**ICC**	**95% CI**	**CV%**	**Mean ± SD**	**ICC**	**95% CI**		**CV%**	**Mean ± SD**	**ICC**	**95% CI**	**CV%**
Distal	Strength Strain Index (mm^3^)	21.7 ± 6.9	0.99	0.98, 1.00	5.6	21.9 ± 5.8	1.00	1.00	1.00	3.5	21.8 ± 6.0	0.99	0.99, 1.00	8.9
Distal	Trabecular area (mm^2^)	200.9 ± 18.8	0.95	0.89, 0.99	2.4	199.9 ± 20.4	0.96	0.93	0.99	2.4	200.4 ± 19.9	0.95	0.89, 0.99	2.4
Distal	Trabecular density (mg.cm^3^)	248 ± 43.1	0.98	0.92, 0.99	3.0	251.9 ± 42.6	0.98	0.97	0.99	2.8	249.9 ± 42.5	0.98	0.91, 0.98	3.0
Mid-shaft	Strength Strain Index (mm^3^)	128.9 ± 41.7	1.00	1.00, 1.00	2.4	130.8 ± 38.4	0.99	0.95	1.00	3.2	129.9 ± 39.8	1.00	0.99, 1.00	2.4
Mid-shaft	Cortical area (mm^2^)	53.4 ± 12.6	1.00	0.99, 1.00	1.5	54.0 ± 11.7	0.99	0.97	1.00	2.4	53.7 ± 12.1	1.00	0.99, 1.00	1.5
Mid-shaft	Cortical density (mg.cm^3^)	1026.5 ± 50.6	0.99	0.97, 1.00	0.1	1024.3 ± 51.7	0.98	0.93	0.99	0.8	1025.2 ± 50.8	0.99	0.95, 0.99	0.5

The most reproducible variable (i.e. lowest precision error) was volumetric cortical bone mineral density with CV% of 0.8% and 0.1% with and without repositioning respectively. The least reproducible variable was strength strain index at the distal metatarsal with CV% ranging from 3.5 (with repositioning) to 8.9 (all scans included). Reproducibility data at the mid-shaft (CV% range 0.1 to 3.2) appeared more reliable than data collected at the distal metatarsal (CV% range 2.4 to 8.9).

We have developed a novel scanning protocol showing acceptable reproducibility for key bone properties measured at the distal and mid-shaft regions of the 2nd metatarsal. Variables selected were considered an appropriate representation of bone geometry (area), bone material (density), and bone strength (strength strain index). All precision values for scans with-and without repositioning were considered to be an indication of good reliability. To our knowledge, we are the first to demonstrate reproducibility of measurements for key bone properties of the 2nd metatarsal using pQCT.

The high reproducibility of volumetric cortical bone mineral density provides potential benefits with regard to fracture prediction. Previous studies have shown fracture failure load in the 2nd metatarsal is strongly dependent on cortical density [8,21]. However we acknowledge that recent research has found that bone geometric strength variables may be more highly correlated with fracture failure loads at the 2^nd^ and 3^rd^ metatarsals than bone density variables [22]. Nevertheless, our results suggest BMD values alone may prove useful in identifying individuals with poor cortical density at the 2^nd^ metatarsal that may be at risk of fracture. Cortical density values acquired at the mid-shaft of the 2^nd^ metatarsal in the current study appeared highly reproducible and may be useful in the early detection of fracture in people with chronic diseases affecting bone health. Indeed cortical area and density measurements at the mid-shaft in the present study were comparable to that of Muehleman et al [8], who used a novel pQCT scanning protocol to measure BMD of fresh frozen, fully disarticulated cadaveric 2^nd^ metatarsal specimens in vitro. In addition, our reproducibility estimates appear to be comparable to that of standard pQCT distal forearm protocols which report CV% values ranging from 0.3-2.2% [23].

One of the potential sources of imprecision with pQCT is patient positioning. Exact repositioning is required to ensure the same region of bone is scanned subsequent to baseline scans. Rigorous positioning control is therefore necessary, particularly if time between re-scans extends to weeks or months. Previous work indicates that small variations in repositioning alters radiation penetration and can result in magnified effects on measurements from repeat scans [11]. In our study, it is plausible that marginally higher CV% values with repositioning occurred as a result of altered limb angles in the gantry and subsequent acquisition of scans at a slightly different plane. It should be noted that all CV% were below 10%, indicating good reliability. We acknowledge that reproducibility estimates for this protocol obtained from live human subjects in future may yield less impressive CV% values.

Previous research has demonstrated repeat scans performed within the same day systematically underestimated precision errors [24]. It is possible that an underestimation of precision errors can lead to a type-II statistical error where a non-significant finding is reported when a true difference actually exists. The current recommendation is to perform repeat scans on different days. Furthermore, the use of different testers has shown increased precision error compared with repeats scans performed by a single tester [24]. In the current study, scans were performed over a two day period by a single tester with similar CV% values with- and without repositioning.

Our reproducibility analysis approach was in accordance with guidelines from the International Society of Clinical Densitometry (ISCD) [12]. The ISCD states that a minimum of 30 degrees of freedom is required through measurement of 30 subjects scanned twice of 15 subjects scanned 3 times to ensure precision error is statistically accurate [25]. In the current study, eleven legs were scanned six times each, representing 55 degrees of freedom ensuring the upper limit for the 95% confidence intervals is no more than 34% greater than the calculated value. The ISCD recommendation for degrees of freedom refers to the calculation of precision errors using DXA. However, it is also recommended that the same procedure is used to determine precision errors for pQCT [12].

The protocol was specifically developed for future clinical application to measure foot bone health in people with chronic inflammatory arthritis conditions such as rheumatoid arthritis who are at risk of metatarsal fractures [26]. However this protocol may have a broader application to other clinical populations with suboptimal bone mass, as well as military recruits, and athletic populations such as gymnasts and runners that are susceptible to fatigue-type stress fractures in the foot [27]. The novel scanning protocol required the construction of a custom-designed acrylic foot plate that allowed the pQCT gantry to pass over the foot scan-region unimpeded. Our results suggest the foot plate provided a stable scanning environment and contributed to the high reproducibility observed in the present study.

There are several limitations in the current study that warrant acknowledgement. We acknowledge that assessments of intra-rater (between-session) and inter-rater agreement – while desirable to establish the reliability of this protocol – were regrettably not possible within the scope and time-frame of this study. Moreover, assessment of accuracy of this protocol via comparison of pQCT outcomes to that of histomorphometric and/or ashing outcomes was also not possible within the scope of this study. Precision values reported in the present study are specific to the testing protocol and therefore may not be representative of different protocols. When scanning parameters, such as voxel size and scan speed, are combined with specific ‘thresholding’ during image analysis, outcome measures are different [28]. Precision values in the current study are specific to the XCT-2000 and may not be representative of other imaging instruments such as XCT-3000 or high resolution pQCT. Furthermore, precision values in the present study are limited to the 2^nd^ metatarsal and cannot be extrapolated to other skeletal sites.

## Conclusions

In conclusion, we have shown excellent reproducibility for key bone variables measured at the distal and mid-shaft regions of the 2^nd^ metatarsal using a novel pQCT scanning protocol. Measurements of cortical density at the mid-shaft of the 2^nd^ metatarsal showed excellent reproducibility (CV < 1.0%). Bone geometry and surrogate markers of bone strength showed acceptable levels of reproducibility. The pQCT protocol will now be applied to measure 2^nd^ metatarsal bone health in children and adults who have inflammatory joint diseases affecting the foot and lower limb, in order to identify those who may be at risk of insufficiency fractures.

## Abbreviations

BMD: Bone mineral density; CV: Coefficient of variation; DXA: Dual x-ray absorptiometry; g.cm^2^: grams per square centimeter; ICC: Intraclass correlation coefficient; ISCD: International Society of Clinical Densitometry; mm^2^: millimeters squared; mm^3^: millimeters cubed; MSE: Mean square error; mSv: millisieverts; pQCT: Peripheral quantitative computed tomography; QRM EFP: Quality Assurance in Radiology and Medicine European Forearm Phantom; ROI: Region of interest; SD: Standard deviation; SSI: Strength strain index; vBMD: Volumetric bone mineral density.

## Competing interests

DJ Schiferl works for Bone Diagnostics Inc (Fort Atkinson, WI, USA).

## Authors’ contributions

GJH was responsible for the original conception of the study and design, acquisition of data, and drafting and revising the manuscript. ST, JB, KR, DS-G, and VdT participated in the design of the study, interpretation of data, and helped to draft the manuscript. DG, EC, DS and AH were responsible for the acquisition of data, data analysis, interpretation of data, and helped to draft the manuscript. All authors read and approved the final manuscript.

## Pre-publication history

The pre-publication history for this paper can be accessed here:

http://www.biomedcentral.com/1471-2474/15/242/prepub
